# Loneliness, Depression, and Inflammation: Evidence from the Multi-Ethnic Study of Atherosclerosis

**DOI:** 10.1371/journal.pone.0158056

**Published:** 2016-07-01

**Authors:** Briana Mezuk, Moon Choi, Amy S. DeSantis, Stephen R. Rapp, Ana V. Diez Roux, Teresa Seeman

**Affiliations:** 1 Department of Family Medicine and Population Health, Division of Epidemiology, Virginia Commonwealth University School of Medicine, Richmond, VA, United States of America; 2 Institute for Social Research, Ann Arbor, MI, United States of America; 3 Korea Advanced Institute for Science and Technology, Daejeon, South Korea; 4 RAND, Santa Monica, CA, United States of America; 5 Department of Psychiatry and Behavioral Medicine, Wake Forest School of Medicine, Winston-Salem, NC, United States of America; 6 Department of Epidemiology and Biostatistics, Dornsife School of Public Health, Drexel University, Philadelphia, PA, United States of America; 7 Division of Geriatrics, David Geffen School of Medicine at the University of California Los Angeles, Los Angeles, CA, United States of America; Cardiff University, UNITED KINGDOM

## Abstract

**Objective:**

Both objective and subjective aspects of social isolation have been associated with alterations in immune markers relevant to multiple chronic diseases among older adults. However, these associations may be confounded by health status, and it is unclear whether these social factors are associated with immune functioning among relatively healthy adults. The goal of this study was to examine the associations between perceived loneliness and circulating levels of inflammatory markers among a diverse sample of adults.

**Methods:**

Data come from a subset of the Multi-Ethnic Study of Atherosclerosis (n = 441). Loneliness was measured by three items derived from the UCLA Loneliness Scale. The association between loneliness and C-reactive protein (CRP) and fibrinogen was assessed using multivariable linear regression analyses. Models were adjusted for demographic and health characteristics.

**Results:**

Approximately 50% of participants reported that they hardly ever felt lonely and 17.2% felt highly lonely. Individuals who were unmarried/unpartnered or with higher depressive symptoms were more likely to report being highly lonely. There was no relationship between perceived loneliness and ln(CRP) (β = -0.051, p = 0.239) adjusting for demographic and health characteristics. Loneliness was inversely associated with ln(fibrinogen) (β = -0.091, p = 0.040), although the absolute magnitude of this relationship was small.

**Conclusion:**

These results indicate that loneliness is not positively associated with fibrinogen or CRP among relatively healthy middle-aged adults.

## Introduction

Loneliness has recently emerged as a novel psychosocial risk factor for a number of health outcomes including cardiovascular disease (CVD). Hawkley, Thisted, Masi, and Cacioppo (2010) reported that loneliness was associated with increased systolic blood pressure longitudinally among middle-aged and older adults [1]. Shiovitz-Ezra and Ayalon (2010) found that loneliness was a risk factor for all-cause mortality among older adults over a 4-year period [2]. Finally, Thurston and Kubzansky (2009) reported a gender difference in the relationship between loneliness and incident coronary heart disease (CHD), such that loneliness was associated with elevated risk of CHD only among women [3].

Although the concept of loneliness shares some commonalities with other aspects of social life, loneliness does not simply mean to be alone [4, 5]. Peplau suggested three main points of agreement across various definitions of loneliness in the existing literature. First, loneliness is a subjective experience distinct from the objective social relationship. Second, loneliness results from a deficiency in a person’s social relationships in terms of type, quality, or quantity relative to perceived need. Finally, the experience of loneliness is *aversive*. In sum, loneliness refers to the sense of perceived social isolation and is defined as a ‘distressing feeling’ that one’s social needs are not met by the quantity or quality of social relationship [4, 5]. Thus, there is a hypothesized disconnect between objective indicators of social life (e.g., network size, frequency of contact) and feeling lonely that can be evaluated empirically.

However, the degree to which the associations between loneliness and health are independent from other psychosocial factors that have linked to CVD and related outcomes, such as social support and depression, is unresolved. Loneliness is negatively associated with poor mental health, likely in a reciprocal fashion [6], and it has been shown that loneliness is strongly associated with depression and increases in depressive symptoms over time [6–9]. Depressive symptoms, in turn, have been associated with both markers of inflammation [10] and CVD risk [11] and there is evidence that these relationships may be bi-directional [12]. There is also evidence that loneliness is related to personality characteristics [13], suicidal behavior [14], and cognitive impairment [15], which are all correlated with depressive symptoms. Thus, efforts to demonstrate loneliness as an independent risk factor for CVD must account for the correlation between perceived social isolation and poor mental health.

Despite epidemiologic evidence of a strong and clinically significant association between loneliness and CVD morbidity and mortality, the biological mechanisms underlying this relationship remain unspecified. In previous work in the Multi-Ethnic Study of Atherosclerosis (MESA) cohort, we have demonstrated that perceived social support has little association with inflammatory markers, either directly or through buffering the negative effects of chronic stress [16]. These findings were contrary to our expectations, and suggested that if social factors are biologically relevant for CVD risk, that aspects of social life other than perceived social support may be driving the relationship. A recent study by Nausheen et al. (2010) reported that loneliness was associated with higher levels of pro-inflammatory cytokines [17]. However this study only recruited cancer patients and thus these findings may not be generalizable to other outcomes or health more generally. Other investigations have reported mixed results about perceived and objective social isolation and markers of inflammation [18–20]. Overall, the association between loneliness and inflammatory markers has not been extensively investigated in population-based studies, including the degree to which loneliness represents a unique predictor of inflammation, independent of depressive symptoms.

Therefore, this study aims to contribute to understanding of how psychosocial factors influence health and illness by examining the associations between perceived loneliness and circulating levels of pro-inflammatory cytokines, specifically C-reactive protein (CRP) and fibrinogen. This study evaluates two hypotheses: (1) perceived loneliness would be associated with higher levels of CRP and fibrinogen; and (2) the association between perceived loneliness would be attenuated, but persist, after accounting for depressive symptoms.

## Materials and Methods

### Sample

Data come from Exam 4 of Multi-Ethnic Study of Atherosclerosis (MESA), an ongoing prospective population-based multi-site study of subclinical CVD started in 2000. Participants were aged 45–84 at baseline with no history of CVD; additional details of the study design are described elsewhere [21]. The MESA sample was free of clinical CVD at baseline, and thus it is well-suited for examining the relationship between loneliness and physiologic changes isolated from the confounding effects of pre-existing CVD that may mask true associations or create spurious ones. While the clinical significance of alterations in these inflammatory markers is not yet fully understood, they may be early indicators of CVD risk. In the Exam 4 (2005–2007) interview (N = 5,818), participants were asked about feelings of perceived loneliness and a subset (N = 456) provided a fasting blood sample for measures of inflammation. We restricted our sample to participants who had complete data on loneliness, CRP and fibrinogen (N = 441, 97% of the eligible subsample).

The MESA was approved by Institutional Review Boards at each site (Columbia University, Johns Hopkins University (JHU), Northwestern University (NWU), University of California–Los Angeles (UCLA), University of Minnesota (UMN), and Wake Forest University). All data were de-identified and analyzed anonymously. This secondary analysis received exempt status from the Institutional Review Board at Virginia Commonwealth University.

### Independent variables

#### Loneliness

Perceived loneliness was measured by three items derived from the UCLA Loneliness Scale [22]: ‘How often do you feel that you lack companionship?’; ‘How often do you feel left out?’ and ‘How often do you feel isolated from others?’ The response categories for each item were 1 = hardly ever, 2 = some of the time, and 3 = often. This short-form of this scale has been used in other large population-based studies [23]. We conducted an exploratory factor analysis and determined that these items described a single factor (Cronbach’s α = 0.79, which is slightly higher than has been reported for other surveys that use the short version of this scale [23]). We then created a summary score (range: 3 to 9) which was mean-centered for analysis. Because of the skewed distribution we also collapsed this summary index into a three-level (0, 1, and 2) categorical variable: Not lonely (score: 3), Moderately lonely (score: 4–5) and Highly lonely (score: 6–9).

### Other covariates

Several demographic characteristics were included in the analysis, including age (in years), sex, race/ethnicity (dichotomized as racial/ethnic minority (Black, Hispanic or Chinese) vs. non-Hispanic white (reference)), educational attainment (dichotomized as high school or less vs. some college or more (reference)), marital status (dichotomized as currently married/partnered vs. not (reference)), and study site (UMN, JHU, Columbia, Wake Forest, NWU and UCLA (reference)). Prevalent hypertension (systolic blood pressure ≥140 mmHg and/or diastolic blood pressure ≥ 90mmHg) and diabetes (fasting glucose >125 mg/dL or use of a hypoglycemic medication) were assessed by clinical examination and combined into a single variable (hypertension and/or diabetes vs. neither (reference)). Poor health behaviors included cigarette smoking (categorized as current vs. former/never), alcohol use (categorized as both whether the participant currently consumed alcohol in the past year, as well as average number of drinks per week among those who did), and body mass index (BMI) in kg/m^2^ calculated from measured height and weight. Current depressive symptoms were assessed using the 20-item Center for Epidemiologic Studies Depression Scale (CESD) [24]; CESD score was treated as both a continuous variable (centered on the mean) and as a categorical variable dichotomized as ≤ 16 vs. >16 to indicate clinically-significant depressive symptoms [25]. We also assessed recent infections (e.g., self-report of cold or flu, sinus infection, urinary tract infection, tooth infection, bronchitis, or pneumonia in the preceding 2 weeks) and use of anti-inflammatory medications (i.e., non-steroidal anti-infiammatory drugs, lipid-lowering medications, hormone therapy, aspirin, and oral anti-inflammatory agents); we conducted sensitivity analyses by refitting our models excluding individuals currently taking these medications or with a recent infection.

### Dependent variables

#### Inflammatory markers

Two inflammatory markers were examined as outcomes: CRP (mg/L) and fibrinogen (mg/dL). CRP is an acute-phase protein and a marker of low-grade systemic inflammation that has been associated with risk of CVD and type 2 diabetes [26, 27]. Fibrinogen helps stop bleeding by promoting the formation of blood clots and has been associated with onset of CVD [27]. Briefly, participants provided fasting venous blood samples, and both high-sensitivity CRP and fibrinogen were assessed using nephelometry (BNII nephelometer and BNII N antiserum to human fibrinogen, respectively, Dade-Behring Inc., San Mateo, CA). Additional details regarding the collection, processing, and storage of the blood samples have been described previously [21].

### Analytic approach

Initially, the relationship between demographic and health characteristics with levels of loneliness was investigated using chi-squared tests for categorical variables and F-tests for continuous variables. The association between loneliness and the two inflammatory markers (hsCRP and fibrinogen) was assessed using multivariable linear regression analyses. We examined loneliness as both a continuous measure and as a three-level categorical measure, as described above. Values of hsCRP and fibrinogen were log-transformed to normalize their distributions for analysis. Regression models were adjusted for age, sex, race/ethnicity, education, hypertension/diabetes status, smoking, alcohol use, BMI and recent infection or use of anti-inflammatory medications.

As part of specifying these regression models we explicitly evaluated the relationship between loneliness and depressive symptoms as indicated by the CESD. Previous investigations have generally excluded the item on loneliness (i.e., ‘I felt lonely’) when calculating the score on the CES-D [4, 6], an approach that artificially separated these two states. We instead empirically investigated this association to determine whether the association between loneliness and inflammation is independent from depression. We ran a series of sensitivity analyses by assessing different specifications of the relationship between depressive symptoms and loneliness: (1) an additive risk factors model in which we included depressive symptoms in our final model as a predictor; (2) a synergistic risk factors model [28] in which we evaluated the interaction between CESD and loneliness; and (3) a competing risk factors model in which we excluded individuals with elevated CESD scores from the analysis. We also ran sensitivity analyses excluding cases of CRP ≥ 10 mg/L (N = 17) and fibrinogen >630 mg/dL (N = 5) to determine if these extreme values influenced out results. Finally, as an additional sensitivity analysis we dichotomized CRP levels using clinically-relevant cut-offs (3mg/L ≤ vs. >3mg/L) [29] and refit the models described above using logistic regression.

All analyses were conducted using STATA v9 and all p-values refer to two-tailed tests.

## Results

Table 1 shows the descriptive characteristics of the analytic sample across the three levels of loneliness. Approximately half (N = 221, 50.1%) of participants did not endorse any feelings of loneliness, and 17.2% were categorized as highly lonely. Being unmarried/unpartnered and higher CES-D scores were positively associated with feelings of loneliness. As expected, there was a strong correlation between CESD scores and loneliness scores (r^2^ = 0.626, p<0.001). CRP and fibrinogen were also positively correlated (r^2^ = 0.539, p<0.001).

**Table 1:** Descriptive Characteristics by Levels of Loneliness.

	Overall	Not lonely	Moderate loneliness	High loneliness	p-value
**N**	441	221	144	76	
**Demographics**					
**Age (M, SD)**	63.4 (8.9)	63.5 (8.4)	63.9 (9.1)	62.0 (10.1)	.312
**Female**	237 (53.7)	108 (48.9)	88 (61.1)	41 (53.9)	.072
**Race/ethnicity**					
**Non-Hispanic White**	205 (46.5)	110 (49.8)	60 (41.7)	35 (46.1)	.526
**Black**	105 (23.8)	52 (23.5)	39 (27.1)	14 (18.4)	
**Hispanic**	91 (20.6)	39 (17.6)	32 (22.2)	20 (26.3)	
**Chinese**	40 (9.1)	20 (9.0)	13 (9.0)	7 (9.2)	
**Married/partnered**	300 (68.0)	172 (77.8)	81 (56.3)	47 (61.8)	< .001
**Education**					
**High school or less**	139 (31.7)	66 (29.9)	49 (34.5)	24 (31.6)	.650
**At least some college**	300 (68.3)	155 (70.1)	93 (65.5)	52 (68.4)	
**Site**					
**WFU**	68 (15.4)	40 (18.1)	23 (16.0)	5 (6.6)	.033
**COL**	82 (18.6)	33 (14.9)	33 (22.9)	16 (21.1)	
**JHU**	59 (13.4)	31 (14.0)	18 (12.5)	10 (13.2)	
**MN**	75 (17.0)	37 (16.7)	20 (13.9)	18 (23.7)	
**NWU**	78 (17.7)	31 (14.0)	29 (20.1)	18 (23.7)	
**UCLA**	79 (17.9)	49 (22.2)	21 (14.6)	9 (11.8)	
**Health behaviors**					
**Smoking status**					
**Current**	49 (11.1)	17 (7.7)	21 (14.6)	11 (14.5)	.073
**Former/Never**	392 (88.9)	204 (92.3)	123 (85.4)	65 (85.5)	
**Currently drink alcohol**					
**Yes**	228 (51.7)	121 (54.8)	70 (48.6)	37 (48.7)	.438
**No**	213 (48.3)	100 (45.2)	74 (51.4)	39 (51.3)	
**# of drinks per week**	0.5 (0.5)	0.6 (0.5)	0.5 (0.5)	0.5 (0.5)	.440
**BMI (kg/m**^**2**^**) (M, SD)**	29.3 (6.1)	29.3 (5.9)	29.3 (6.1)	29.6 (6.5)	.923
**Mental health**					
**CESD score (M, SD)**	8.2 (8.4)	4.2 (3.9)	9.5 (7.7)	17.5 (10.9)	< .001
**CESD≤ 16**	373 (84.6)	221 (100)	115 (79.9)	37 (48.7)	< .001
**CESD>16**	68 (15.4)	0	29 (20.1)	39 (51.3)	
**Health status**					
**Hypertension or diabetes**	231 (52.4)	111 (50.2)	84 (58.3)	36 (47.4)	.200
**Recent infection**	90 (20.4)	39 (17.6)	36 (25.0)	15 (19.7)	.231
**Anti-inflammatory medication use**	264 (59.9)	126 (57.0)	93 (64.6)	45 (59.2)	.351
**Inflammatory markers**					
**Ln(hsCRP) (M, SD)**	0.51 (1.04)	0.55 (1.05)	0.43 (1.07)	0.55 (0.94)	.578
**Ln(Fibrinogen) (M, SD)**	5.92 (0.20)	5.92 (0.20)	5.94 (0.20)	5.88 (0.22)	.089

Values are N (%) unless otherwise noted. P-value from chi-squared tests for categorical variables and F-tests for continuous variables.

As shown by Tables 2 and 3, there was no association between feelings of loneliness, measured as either a continuous score or a categorical variable, and CRP, either in unadjusted models or after accounting for demographic characteristics and risk factors. Analyses excluding cases of elevated CRP produced similar results (Tables A and B in the S1 File). Contrary to our hypothesis, higher levels of loneliness were associated with lower levels of fibrinogen, although associations were of small magnitude (Tables 2 and 3). These associations were similar for men and women in analyses stratified by gender (Table C in the S1 File).

**Table 2:** Association between loneliness and inflammatory markers.

	Ln(CRP)	Ln(Fibrinogen)
	β (SE), p-value	β (SE), p-value
**Loneliness**	-0.03 (0.03),0.269	-0.01 (0.01), 0.043
**Age**	0.01 (0.01),0.109	0.01 (0.01), <0.001
**Female**	-0.14 (0.09),0.122	-0.08 (0.02), <0.001
**Racial/ethnic minority**	0.11 (0.11),0.305	0.03 (0.02), 0.120
**Married/partnered**	0.11 (0.10),0.265	-0.02 (0.02), 0.327
**More than high school education**	0.09 (0.10),0.379	0.01 (0.02), 0.776
**Current smoker**	0.42 (0.14),0.004	0.05 (0.03), 0.097
**Current drinker**	-0.06 (0.10),0.552	-0.03 (0.02), 0.090
**BMI**	0.09 (0.01),<0.001	0.01 (0.01), <0.001
**Prevalent hypertension or diabetes**	-0.16 (0.09),0.094	-0.01 (0.02), 0.717
**N**	441	441
**Adjusted R**^**2**^	0.24	0.21

Estimates are adjusted for study site, current use of anti-inflammatory medications, and recent infection. Racial/ethnic minority includes African American, Hispanic, and Chinese.

**Table 3:** Association between categories of loneliness and inflammatory markers.

	Ln(CRP)	Ln(Fibrinogen)
	β (SE), p-value	β (SE), p-value
***Reference*: *Not lonely***		
**Moderate loneliness**	-0.16 (0.10), 0.123	-0.01 (0.02), 0.869
**High loneliness**	-0.07 (0.12), 0.578	-0.06 (0.02), 0.013
**N**	441	441
**Adjusted R**^**2**^	0.25	0.21

Estimates are adjusted for age, sex, race/ethnicity, marital status, site, smoking status, drinking status, education, BMI, prevalent hypertension or diabetes, recent infection, and current use of anti-inflammatory medication.

We then examined the relationship between depressive symptoms, loneliness, and the inflammatory markers. In models that included CESD score as an additional covariate (additive model), neither CESD nor loneliness were significantly associated with either CRP or fibrinogen (Tables D and E in the S1 File). Next we tested the synergistic model. In unadjusted models the interaction term between the continuous measures of CESD and loneliness was null for CRP (β: 0.0003, p = 0.934) and small but statistically significant for fibrinogen (β: -0.001, p = 0.024); however, after adjusting for demographic and health characteristics the interaction was no longer statistically significant (β: -0.001, p = 0.408). Finally we evaluated the competing risks model of depression and loneliness (Table F in the S1 File). After excluding individuals with CESD>16 (N = 68), the continuous measure of loneliness was significantly associated with lower lnCRP (β: -0.10, 95% Confidence Interval (CI): -0.18, -0.01, p = 0.028). Similarly, in this restricted sample loneliness was marginally associated with lower fibrinogen (β: -0.01, 95% CI: -0.03, 0.002, p = 0.097).

We repeated these analysis using clinically-elevated CRP (>3mg/L) as the outcome. Approximately 28% of the sample (N = 117) had elevated CRP. In the additive model, loneliness was not significantly associated with CRP (Odds ratio (OR): 0.84, 95% CI: 0.67–1.06). There was also no evidence that loneliness and depression acted synergistically (OR_interaction_: 1.01, p = 0.956), consistent with the linear regression results. Finally, after excluding individuals with CESD>16, loneliness was significantly associated with lower relative odds of high CRP (OR: 0.70, 95% CI: 0.53–0.92), consistent with the analysis of lnCRP.

## Discussion

The primary finding from this study is that in this relatively healthy sample of middle-aged and older adults loneliness is not associated with higher levels of inflammatory markers. Contrary to our expectations, there was no evidence of a positive relationship between feelings of loneliness and these two markers of inflammation. Finally, although depressive symptoms and feelings of loneliness were highly correlated and have both been associated with elevated levels of inflammatory markers in prior studies, these states did not interact in a synergistic fashion in our sample. In contrast, there was suggestive evidence that the inverse relationship between loneliness and inflammation was stronger among those without elevated depressive symptoms.

Our findings do not support the hypothesis that the observed epidemiologic relationship between loneliness and health is mediated through inflammatory pathways. Although many previous studies have reported a significant positive association between loneliness and inflammatory markers [17, 30, 31], others have failed to replicate this finding [18, 32]. It may be that the relationship between loneliness and biological changes is an indirect one, such that loneliness does not affect biological parameters directly but rather enhances the effects of other psychosocial stressors (which have themselves been associated with biological changes [31, 33, 34]. Some reports suggest that loneliness is associated with inflammatory markers through a more indirect pathway by moderating the body’s biological response to acute stress [30, 31]. It is important to acknowledge that levels of inflammatory markers assessed from a single venipuncture do not fully capture the dynamic processes involved in inflammation, or allow us to distinguish acute from chronic inflammation. For example, chronically elevated IL-6 levels have been associated with psychological disorder (particularly depression [35]) and changes in immune markers CRP and cortisol have been associated with loneliness longitudinally [36]. Also, recent studies examining expression of immune-related genes in leukocytes suggest that loneliness is associated with upregulation of pro-inflammatory genes [37], and these types of measures may be more sensitive indices of the biological correlates of psychological states like loneliness [38].

These results should be interpreted in light of the strengths and limitations of the study. MESA is a population-based sample of individuals initially free of prevalent CVD; this provides an opportunity to examine the relationship between psychosocial characteristics and pre-clinical biological markers of CVD risk without confounding by pre-existing health conditions. The MESA clinical assessment protocols were followed with high fidelity, and the cohort as whole has experienced only minimal loss to follow-up. We were also able to account for a range of health characteristics known to influence inflammatory markers, such as tobacco use, BMI, and alcohol use. This study also has limitations. Because loneliness was not measured at prior interviews, we were only able to examine its relationship with inflammation in a cross-sectional manner which precludes any interpretation of causal effects. Only a subsample of the Exam 4 participants provided a blood sample for analysis, and the two markers we examined may not be the indicators of systematic inflammation most sensitive to psychosocial factors like loneliness. Loneliness was assessed at only one point in time when participants were approximately 63 years old; it may be that loneliness has more relevance to these biomarkers at other points in the life course or that chronic experiences of loneliness are more relevant to health than our static assessment. Finally, our abbreviated measure of loneliness may not have captured other relevant aspects of this state, such as feelings of hopelessness or apathy.

In sum, these findings add to the growing body of literature aimed at understanding the mechanisms by which factors such as social isolation and perceived loneliness may influence health in later life. Although our results do not support inflammation as a general pathway linking loneliness and health, other biological or behavioral mechanisms may still be relevant. It may also be that chronic loneliness, rather than acute loneliness assessed at one point in time, is more relevant to these biomarkers. Finally, emerging research indicates that low levels of loneliness, much like high levels of social support, may buffer the effect of stressors on health; future studies should continue to explore both the direct and indirect pathways that may explain the observed associations between loneliness and health in later life.

## Supporting Information

S1 File Sensitivity analyses described in the text (Tables A-F).
(DOCX)
