# Supplementary material for: Loneliness, Depression, and Inflammation: Evidence from the Multi-Ethnic Study of Atherosclerosis
Source: PLoS One. 2016 Jul 1;11(7):e0158056. doi: 10.1371/journal.pone.0158056 (PMC4930171; doi:10.1371/journal.pone.0158056)
Supplement: S1 File — Sensitivity analyses described in the text (Tables A-F). (DOCX) [file pone.0158056.s001.docx]

Supplementary file 1

**Table A: Association between loneliness and inflammatory markers excluding high values of CRP and fibrinogen**

|  | **Ln(CRP)** | **Ln(Fibrinogen)** |
| --- | --- | --- |
|  | **β (SE), p-value** | **β (SE), p-value** |
| Loneliness | -0.03 (0.03), 0.342 | -0.01 (0.01), 0.050 |
| Age | 0.01 (0.01), 0.227 | 0.01 (0.01), <0.001 |
| Female | -0.11 (0.09), 0.229 | -0.07 (0.02), <0.001 |
| Racial/ethnic minority | 0.06 (0.11), 0.599 | 0.04 (0.02), 0.041 |
| Married/partnered | 0.13 (0.10), 0.166 | -0.02 (0.02), 0.417 |
| More than high school education | 0.09 (0.10), 0.373 | 0.02 (0.02), 0.352 |
| Current smoker | 0.10 (0.14), 0.004 | 0.04 (0.03), 0.128 |
| Current drinker | -0.06 (0.09), 0.505 | -0.03 (0.02), 0.096 |
| BMI | 0.08 (0.01), <0.001 | 0.01 (0.01), <0.001 |
| Prevalent hypertension or diabetes | -0.17 (0.09), 0.062 | -0.01 (0.02), 0.554 |
| N | 424 | 436 |
| Adjusted R^2^ | 0.22 | 0.19 |

Estimates are adjusted for study site, current use of anti-inflammatory medications, and recent infection.

Racial/ethnic minority includes African American, Hispanic, and Chinese.

**Table B: Association between categories of loneliness and inflammatory markers, excluding high values**

|  | **Ln(CRP)** | **Ln Fibrinogen** |
| --- | --- | --- |
|  | **β (SE), p-value** | **β (SE), p-value** |
| *Reference: Not lonely* |  |  |
| Moderate loneliness | -0.14 (0.10), 0.153 | -0.01 (0.02), 0.739 |
| High loneliness | -0.05 (0.12), 0.657 | -0.06 (0.02), 0.021 |
| N | 424 | 436 |
| Adjusted R^2^ | 0.22 | 0.19 |

Estimates are adjusted for age, sex, race, marital status, site, smoking status, drinking status, education, BMI, prevalent hypertension or diabetes, recent infection, and current use of anti-inflammatory medication.

**Table C: Association between loneliness and inflammatory markers stratified by sex**

|  | **Ln(CRP)** | **Ln(Fibrinogen)** |
| --- | --- | --- |
|  | **β (SE), p-value** | **β (SE), p-value** |
| **Female** |  |  |
| Loneliness | -0.07 (0.09), 0.428 | -0.03 (0.02), 0.108 |
| N | 237 | 237 |
| Adjusted R^2^ | 0.37 | 0.19 |
| **Male** |  |  |
| Loneliness | -0.11 (0.09), 0.202 | -0.03 (0.02), 0.042 |
| N | 204 | 204 |
| Adjusted R^2^ | 0.08 | 0.12 |

Estimates are adjusted for age, race/ethnicity, marital status, site, education, smoking status, drinking status, BMI, having hypertension or diabetes, recent infection, and current use of anti-inflammatory medication.

**Table D: Association between loneliness and inflammatory markers, excluding participants with CESD>16**

|  | Ln(CRP) | Ln(Fibrinogen) |
| --- | --- | --- |
|  | β (SE), p-value | β (SE), p-value |
| Loneliness | -0.10 (0.04), 0.028 | -0.01 (0.01), 0.097 |
| N | 373 | 373 |
| Adjusted R^2^ | 0.25 | 0.21 |

Estiamtes are adjusted for age, sex, race/ethnicity, marital status, site, smoking status, drinking status, education, BMI, prevalent hypertension or diabetes, recent infection, and current use of anti-inflammatory medication.

**Table E: Association between loneliness and depressive symptoms with inflammatory markers**

|  | **Ln CRP** | **Ln Fibrinogen** |
| --- | --- | --- |
|  | **β (SE), p-value** | **β (SE), p-value** |
| Loneliness | -0.04 (0.04), 0.361 | -0.01 (0.01), 0.463 |
| Depressive symptoms | 0.001 (0.007), 0.951 | -0.002 (0.001), 0.148 |
| N | 441 | 441 |
| Adjusted R^2^ | 0.25 | 0.21 |

Estimates are adjusted for age, sex, race/ethnicity, marital status, site, smoking status, drinking status, education, BMI, prevalent hypertension or diabetes, recent infection, and current use of anti-inflammatory medication.

**Table F: Association between loneliness and elevated (CESD>16) depressive symptoms with inflammatory markers**

|  | Ln(CRP) | Ln(Fibrinogen) |
| --- | --- | --- |
|  | β (SE), p-value | β (SE), p-value |
| Loneliness | -0.05 (0.04), 0.150 | -0.01 (0.01), 0.311 |
| Elevated depressive symptoms | 0.138 (0.15), 0.346 | -0.038 (0.03), 0.198 |
| N | 441 | 441 |
| Adjusted R^2^ | 0.24 | 0.21 |

Estimates are adjusted for age, sex, race/ethnicity, marital status, site, smoking status, drinking status, education, BMI, prevalent hypertension or diabetes, recent infection, and current use of anti-inflammatory medication.
